# 1281. Ongoing Transmission of Clonal Strains of Invasive Nontypeable *Haemophilus influenzae* Disproportionately Affecting Persons with HIV in Metropolitan Atlanta, 2019—2022

**DOI:** 10.1093/ofid/ofad500.1120

**Published:** 2023-11-27

**Authors:** Lauren F Collins, Samantha Sefton, Stepy Thomas, Amy Tunali, Andrei Bombin, Timothy D Read, Sarah W Satola, Monica M Farley

**Affiliations:** Emory University School of Medicine, Division of Infectious Diseases, Atlanta, Georgia; Emory University, Brookhaven, Georgia; Emory University, Brookhaven, Georgia; Emory University, Brookhaven, Georgia; Emory University School of Medicine, Atlanta, Georgia; Emory University School of Medicine, Atlanta, Georgia; Emory University School of Medicine, Division of Infectious Diseases, Atlanta, Georgia; Emory University School of Medicine, Division of Infectious Diseases, Atlanta, Georgia

## Abstract

**Background:**

Nontypeable *Haemophilus influenzae* (NTHi) is a genetically diverse pathogen that typically causes mucosal infections or invasive NTHi (iNTHi) disease in persons at the extremes of age. In 2017-2018, we reported a significant increase in incident iNTHi in Atlanta among virally-suppressed persons with HIV (PWH) and identified two clonal strains associated with septic arthritis/polyarthritis among Black men who have sex with men.

**Methods:**

Surveillance data from the CDC-funded Georgia Emerging Infections Program was used to assess interim iNTHi epidemiology in adults ≥ 18 years old in metropolitan Atlanta, including the potential for continued transmission of clonal strains in adults 18-55 years old. Overall incidence of iNTHi and by HIV status from 2019-2022 was determined and compared with prior years; and demographic and clinical data were collected. Whole genome sequencing assessed genetic relatedness of isolates, which were categorized as belonging to cluster 1 or 2 (C1, C2) or non-clustered (NC).

**Results:**

From 2019-2022 in Atlanta, there were 157 iNTHi cases in adults ≥ 18 years old (median age 56 [Q1-Q3 36-72] years, 59% male, 44% Black), including 29 in PWH. The iNTHi incidence among PWH in 2019-2022 was lower than in 2017-2018 (18.8 vs 41.7 cases per 100,000, respectively, *p*< 0.01), and higher than in 2008-2016 (9.6 per 100,000; *p*< 0.01) (Figure). Among iNTHi cases 18-55 years old (2019-2022), 94/95 had isolates available for cluster analysis: 7/94 (7%) and 18/94 (19%) were identified as C1 and C2, respectively. Characteristics of cases differed by clonal status (Table 1): among C1, C2, and NC cases, 6/7 (86%), 7/18 (39%), and 15/69 (22%) involved PWH, respectively; and septic arthritis was present in 1/7 (14%), 8/18 (44%), and zero, respectively. Table 2 provides detailed data on PWH with clonal strains.

**Figure:**
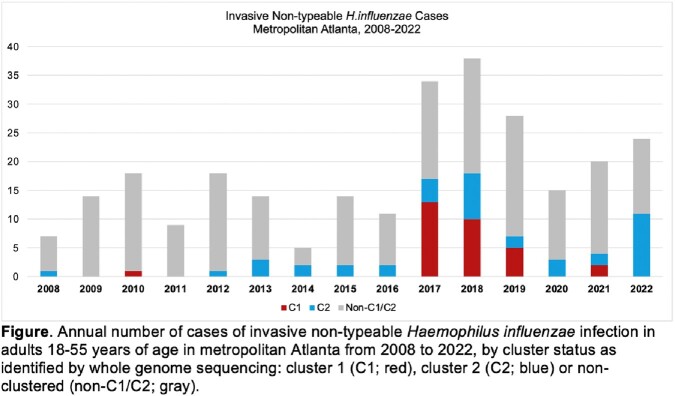

**Figure:**
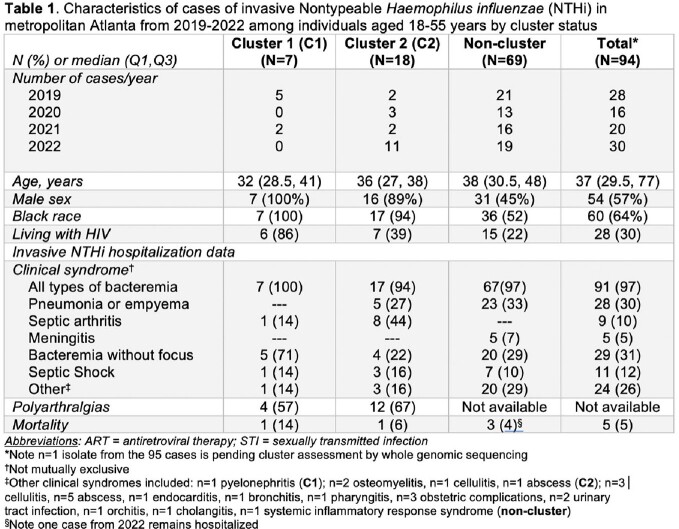

**Figure:**
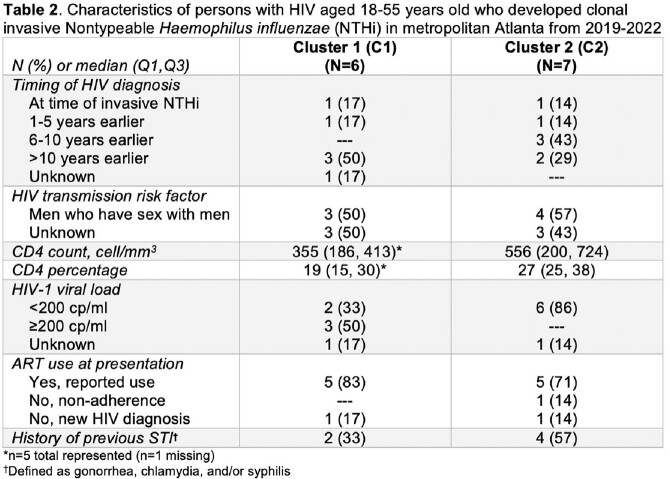

**Conclusion:**

Ongoing transmission of two clonal NTHi strains that are associated with a relatively high prevalence of joint involvement in invasive disease and disproportionately affect Black men and PWH was noted in Atlanta. The C2 clonal strain became more prevalent in 2022. Additional studies are needed to better understand modes of transmission, geographic distribution of clones, and to explore the unusual clinical manifestations to optimize prevention and treatment measures.

**Disclosures:**

**Lauren F. Collins, MD, MSc**, Curio Science: Honoraria

